# Stable Oxygen Incorporation in Superconducting TaN: An Experimental and Theoretical Assessment

**DOI:** 10.1021/acsomega.4c05310

**Published:** 2024-08-01

**Authors:** Victor Quintanar-Zamora, Michelle Cedillo-Rosillo, Oscar Contreras-López, Carlos Antonio Corona-Garcia, Armando Reyes-Serrato, Rodrigo Ponce-Pérez, Jonathan Guerrero-Sanchez, Jesús Antonio Díaz

**Affiliations:** † Posgrado en Nanociencias, Centro de Investigación Científica y de Educación Superior de Ensenada, Ensenada, Baja California 22860, Mexico; ‡ Centro de Nanociencias y Nanotecnología, Universidad Nacional Autónoma de México, Ensenada, Baja California 22860, Mexico

## Abstract

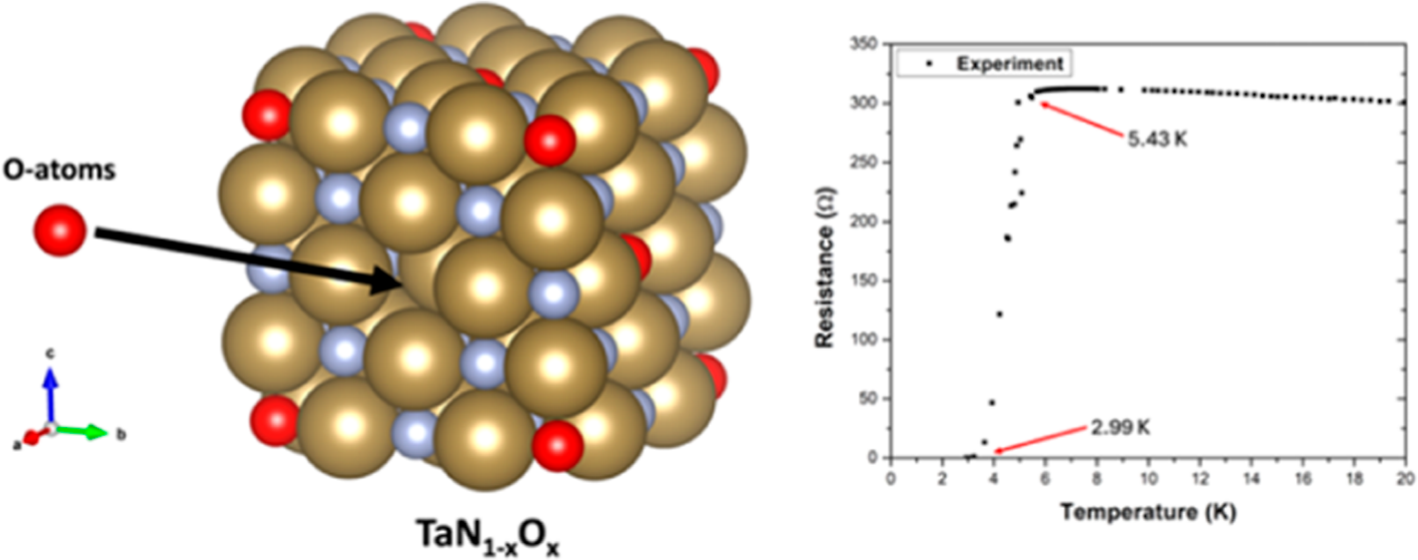

Oxide formation in superconducting TaN thin films is analyzed through experimental measurements and computational simulations. TaN was synthesized in an ultrahigh vacuum (UHV) system by reactive pulsed laser deposition and characterized *in situ* by X-ray photoelectron spectroscopy; it was also characterized *ex situ* by X-ray diffraction, transmission electron microscopy, and the four-point probe method. Despite being grown in an UHV chamber with a base pressure of 5 × 10^–9^ Torr, TaN contains a significant amount of oxygen (up to 20 at. %) attributed to residual gases containing O atoms. Several TaN_1–*x*_O_*x*_ models, with different amounts of O atoms incorporated into N sites, were simulated using *ab initio* calculations to assess the feasibility of oxide formation. Thermodynamic stability analysis reveals that TaN_1–*x*_O_*x*_ stability increases with oxygen addition, indicating that its incorporation is thermodynamically favorable. The oxygen-impurified TaN exhibits a face-centered cubic structure and is a superconductor (*R* = 0 Ω) at 2.99 K. The results discussed here highlight the importance of considering stable oxygen impurities when studying superconductivity in TaN films. The formation of TaN_1–*x*_O_*x*_ regions in the compound may be key to understanding the variation in critical temperature reported in the literature.

## 1 Introduction

Tantalum nitride is a transition metal nitride that presents a wide variety of crystalline phases such as β-TaN_0.05_, γ-Ta_2_N, δ-TaN_1–*x*_, ε-TaN, Ta_5_N_6_, Ta_4_N_5_, and Ta_3_N_5_.^1−4^ Within their different polymorphs, only δ-TaN_1–*x*_ (from now on TaN) exhibits superconductivity at ambient pressure at a maximum critical temperature (*T*_c_) of 10.8 K.^5^ This compound crystallizes in a NaCl-type structure with a lattice parameter of 4.34 Å.^6,7^ Despite being metastable, several groups have reported its thin film growth by reactive sputtering,^2,5,8−10^ and laser ablation.^11^

Superconducting TaN has recently attracted attention due to its applications in the detection of low-energy photons in superconducting nanowire single-photon detectors,^12^ in the development of metal–insulator–superconductor (MIS) tunnel junctions,^13^ in superconducting spintronics due to its quasiparticle-mediated inverse spin Hall effect, which can be used to probe spin currents in emergent quantum materials,^14^ or in spin–orbit torques (SOTs) for upcoming topological electronics.^15^

Superconductivity in TaN was first measured in thin films (*T*_c_ = 4.84 K)^2^ and then in bulk (*T*_c_ = 6.5 K).^16^ Various authors have reported superconducting TaN thin films *T*_c_ within a wide range from 0.5 to 10.8 K.^5,8−11^ This *T*_c_ variation has been mainly attributed to the nitrogen content in the film;^5,8^ however, these studies have not considered possible carbon or oxygen impurities. It should be noted that superconductivity in polycrystalline face-centered cubic (FCC) TaO bulk was recently reported.^17^ Therefore, Ta–O impurities in TaN thin films are expected to be superconducting if the FCC structure is conserved.

Other studies, unrelated to superconductivity, point out that oxygen is consistently detected by X-ray photoelectron spectroscopy (XPS) at the surface of TaN thin films. For instance, TaN films grown in an ultrahigh vacuum (UHV) chamber and analyzed *in situ* present an oxygen concentration between 2 and 5% after high ion doses, and no other surface contaminants, such as carbon, are detected.^18^ Additionally, Ta sheets irradiated under a high vacuum or in the presence of N_2_ exhibit a measurable oxygen concentration at the sample surface, regardless of the gas phase composition used during the laser treatment.^19^

Cristea *et al.* reported the growth of FCC tantalum oxynitride (TaO_*x*_N_*y*_) thin films by DC reactive magnetron sputtering.^20^ They characterized its composition and structure by Rutherford backscattering spectrometry and X-ray diffraction (XRD), respectively, demonstrating that oxygen in FCC TaO_*x*_N_*y*_ films can exist as an oxynitride. Oxynitrides exhibit a wide range of compositions,^21^ and their physical properties are sensitive to the nitrogen/oxygen ratio;^22^ tuning this ratio can lead to potentially attractive properties.^23^ These materials have received attention for their diverse applications, including pigments,^24^ water-splitting,^25^ corrosion resistance,^26^ antibacterial properties, and photocatalytic activity.^27^

This manuscript presents experimental and theoretical evidence of non-negligible oxygen content in superconducting TaN. The superconductive nature of the oxygen-impurified TaN thin films remains (*T*_c_ = 2.99 K), even at an oxygen content of 20 at. % incorporated into the FCC lattice. An atomic-scale analysis of various FCC TaN_1–*x*_O_*x*_ (*Fm*3̅*m*) models using DFT calculations together with a thermodynamic stability criterion revealed that O atoms substitute N atoms from TaN without structural phase modifications and with minimal changes in the lattice parameter, augmenting its stability as the oxygen content increases. Several studies on superconducting FCC TaN have appeared; however, the properties of its TaN_1–*x*_O_*x*_ phases have yet to be reported.

## 2 Methods

### 2.1 Experimental Procedure

The TaN thin films were synthesized by reactive pulsed laser deposition (PLD) in a modified RIBER LDM 32 UHV system. A 99.99% pure tantalum target from Kurt J. Lesker was employed as a source of Ta atoms. A 6 ns and 266 nm wavelength pulse from a Surelite Continuum Nd:YAG solid-state laser was used to ablate the target. The laser has a 1064 nm fundamental wavelength, a second harmonic at 533 nm, and a fourth harmonic at 266 nm. The working pressure of the chamber was set to 0.09 Torr of ultrahigh purity N_2_ gas (99.999%). Then, a 35 nm thick tantalum nitride thin film was deposited on an FCC MgO (100) substrate from Sigma-Aldrich to achieve epitaxial growth at 850 °C for 220 min. The size of the film is 1 cm long × 0.5 cm wide × 35 nm thick.

The UHV system is composed of three chambers separated by gate valves. First, the introduction chamber enables the target and substrates to be placed inside the UHV system without exposing the other chambers to ambient pressure; its base pressure is 1.2 × 10^–8^ Torr. Next, the growth chamber permits the production of thin films by reactive PLD; its base pressure is 5 × 10^–9^ Torr. Finally, the analysis chamber allows measuring *in situ* the chemical composition of the thin films by XPS; its base pressure is 1.2 × 10^–9^ Torr. The passage of samples from one chamber to another is conducted by a transporting bar. This system prevents the thin film from being exposed to ambient pressure before measurement by *in situ* XPS.

Before the target and substrates entered to the UHV system, it was baked for 48 h to degas the internal walls of the chambers. Additionally, the N_2_ gas line was purged for 24 h. Then, the gas pumping valve was closed, and a vacuum of 5 × 10^–9^ Torr was reached in the growth chamber. After that, the target and substrates, which had been cleaned with ultrasound and acetone, were introduced into the UHV system. Before each deposition, to eliminate surface contaminants, the target was cleaned by laser ablation for 5 min, and the MgO substrate was heated to 250 °C for 12 h, both under UHV conditions.

The chemical composition was analyzed by *in situ* XPS at a pressure of 1.2 × 10^–9^ Torr and at room temperature. A depth profile for the thin film was not performed. All other characterizations were performed *ex situ* at standard conditions. The TaN thin film was not capped for any of the measurements. The XPS spectra were registered immediately after deposition with a SPECS PHOIBOS hemispherical analyzer, where the X-rays were produced with a monochromatic source of Al Kα (1486.6 eV). First, a low-resolution spectrum was measured at a pass energy of 100 eV and a dwell time of 0.1 s to identify the elemental components of the film. Next, the high-resolution spectra of the Ta 4f, N 1s, and O 1s peaks were measured at a pass energy of 10 eV and a dwell time of 0.5 s to quantify the previously identified components; the Ta 4p_3/2_ peak was also measured because it overlaps with the N 1s peak. The background noise was subtracted using the Shirley method,^28^ and the peak area of each component was calculated separately. The elemental concentration (*C*) in atomic percentage (at. %) was calculated using the following equation
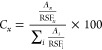
1where *A* is the peak area and RSF is the relative sensitivity factor. In addition, the Ta chemical environment was identified from the curve fitting of the Ta 4f peak. The NIST Standard Reference Database 20^29^ was used as a reference for peak identification. All the spectra were processed with CasaXPS software.^30^

The crystal structure was subsequently identified by XRD with a PANalytical X’Pert Pro MRD diffractometer in the 2θ range from 30 to 80° using a glancing angle of 0.1°, a step of 0.02°, and a data acquisition time of 0.5 s. The X-rays were produced by a Cu Kα source (λ = 1.5406 Å). The sample reflections were indexed using the ICDD database. The data were processed with HighScore Plus software.^31^ In addition, the local crystal structure was explored with a JEOL JEM-2100F scanning transmission electron microscope (STEM), which has a Schottky-type field emission electron gun (FEG) with a 200 kV electron beam corresponding to a wavelength of approximately 0.0025 nm. The specimen was analyzed in conventional parallel-beam mode. Then, high-resolution micrographs of the cross-section of the thin film and its electron diffraction pattern were recorded. The sample was prepared with a JEOL JIB-4500 scanning electron microscope focused ion beam to reduce the film cross-sectional thickness to less than 100 nm by micromilling with gallium ions. The micrographs and diffraction patterns were processed with DigitalMicrograph software.

The resistance *vs* temperature curve was measured by the four-point probe method in a Quantum Design DynaCool physical properties measurement system to determine the superconducting transition temperature. The sample was cooled from room temperature to 2 K and measured at a constant direct current of 0.05 mA.

### 2.2 DFT Calculations

Atomic-scale description of TaN_1–*x*_O_*x*_ (0 ≤ *x* ≤ 1) was performed with the Vienna *ab initio* simulation package (VASP) code.^32−37^ The electron–ion interactions were treated by employing projector augmented wave pseudopotentials^38^ with an energy cutoff of 500 eV. The exchange–correlation energy was described by the generalized gradient approximation with the Perdew–Burke–Ernzerhof (PBE) parametrization.^39^ The Brillouin zone was sampled using the special point scheme of Monkhorst–Pack^40^ with a *k*-point grid of 8 × 8 × 8 for the unit cell. During geometry optimization, convergence is achieved when the energy differences are less than 1.0 × 10^–4^ eV and all the force components are less than 0.01 eV/Å. Oxygen concentrations were treated using the supercell method, with a 2 × 2 × 2 periodicity.

Since we are treating different oxygen concentrations, the total energy of these structures is not a good parameter to determine the models’ stability. Instead, the defect formation energy (DFE) formalism,^41^ which is independent of the number of atoms, is a good parameter for determining stable systems. To apply the DFE formalism, a thermodynamic equilibrium is considered between TaN and its constituents, implying the following
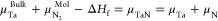
2where μ_*i*_ is the chemical potential of the *i*th species and Δ*H*_f_ is the enthalpy of formation of TaN. The calculated Δ*H*_f_ is −1.70 eV/atom, which is in agreement with previous reports.^42,43^ For a system composed of Ta, N, and O, the DFE takes the following form
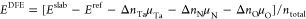
3where *E*^slab^ is the total energy of the system at hand, *E*^ref^ is the total energy of an arbitrary reference, which in this case is the pristine TaN, Δ*n*_*i*_ is the excess or deficit of *i*th atoms with respect to the reference, and *n*_total_ is the total number of atoms in the system. In this analysis, all atomic configurations have the same number of Ta atoms, while the N and O atoms vary. Therefore, we vary the chemical potential to evaluate the DFE in a range from O-rich conditions () to O-poor conditions (), where *E*^cohesive^ is the cohesive energy for the oxygen atoms.

## 3 Results and Discussion

### 3.1 Chemical Composition

**Figure 1:**
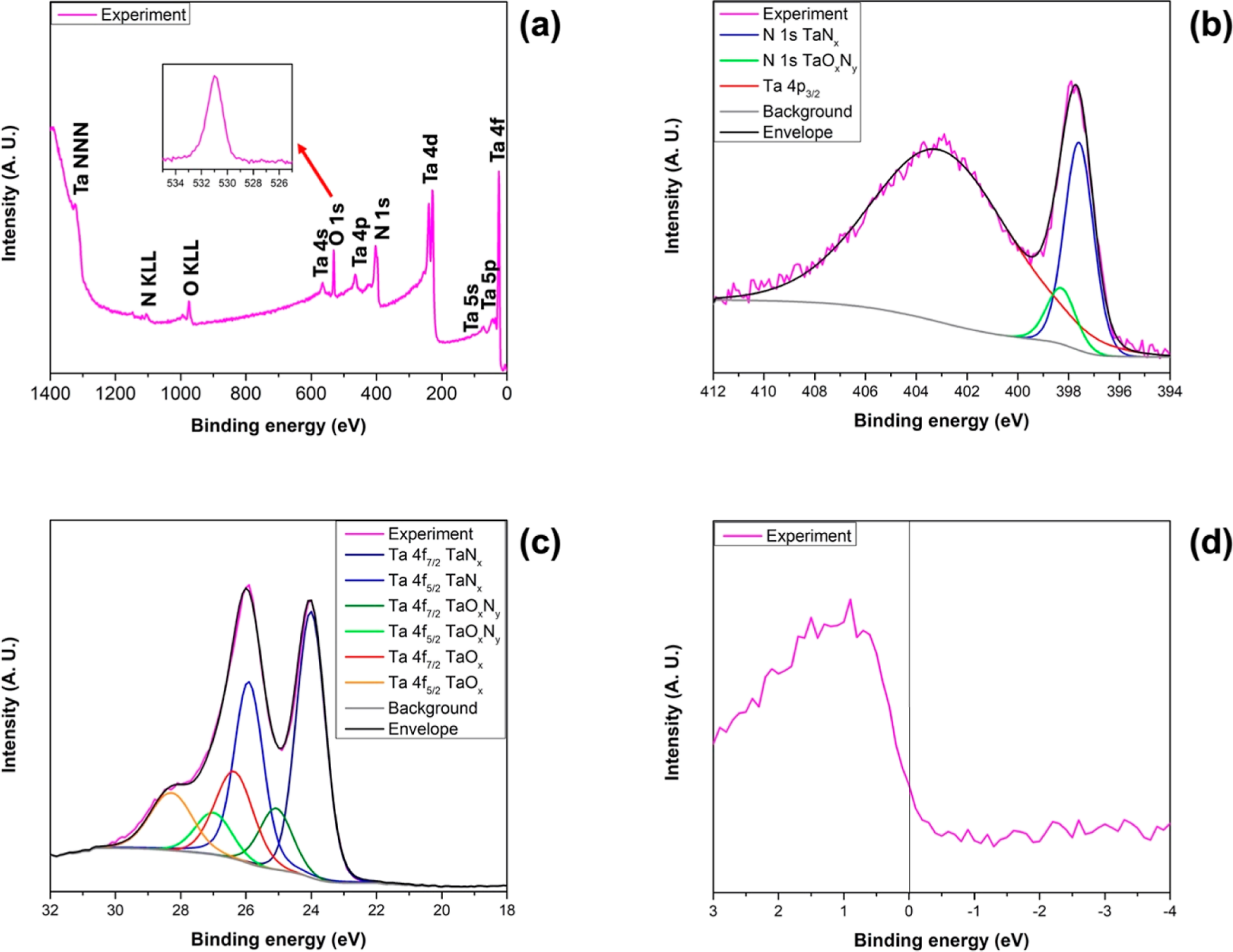
Chemical analysis of the thin film by XPS. (a) Survey low-resolution spectrum and O 1s high-resolution spectrum, (b) N 1s and Ta 4p_3/2_ high-resolution spectrum, (c) Ta 4f high-resolution spectrum, and (d) valence band region high-resolution spectrum.

The chemical components of the thin film were identified from the XPS survey spectrum shown in Figure 1a, in which its binding energy ranges from 0 to 1400 eV; the appearance of several characteristic peaks such as Ta 4f, N 1s, and O 1s reveals that the sample does not contain any other impurities besides O. The C 1s peak is not found, even though carbon is a common contaminant. The inset in Figure 1a also shows the high-resolution spectrum of O 1s from 525 to 535 eV, where its maximum binding energy appears at 531.2 eV. The peak area was obtained in the CasaXPS program after the background noise subtraction. Note that oxygen was not intentionally introduced into the system. Therefore, oxygen acts as a stable impurity in the sample, located on the surface and throughout the material since the sample was isolated from the environment before and during analysis. Oxygen appeared even though the film was deposited with a controlled pressure of ultrahigh purity N_2_ due to the residual gases in the UHV chamber containing O atoms.

Figure 1b shows the high-resolution spectrum of the region containing the Ta 4p_3/2_ and N 1s peaks from 394 to 412 eV, where the maximum binding energy appears at 397.9 eV; it also shows the curve fitting of each peak separately, in which the N 1s associated with tantalum nitride is at 397.6 eV, and the N 1s associated with tantalum oxynitride is at 398.3 eV; on the other hand, Ta 4p_3/2_ is located at 403.1 eV. The envelope exhibits a good fit with the experimental data. The magenta line in the graph corresponds to the experimental data, the blue line to the N 1s associated with TaN_*x*_, the green line to the N 1s associated with TaO_*x*_N_*y*_, the red line to the Ta 4p_3/2_, the gray line to the background, and the black line to the fitting envelope. The shift to higher binding energies of the Ta 4p_3/2_ signal confirms the formation of the Ta–N bond since the signal for metallic Ta is reported at 400.9 eV^44^ and for TaN at 403.0 eV.^45^

Figure 1c shows the high-resolution spectrum of the Ta 4f peak from 18 to 32 eV. The spectrum exhibits the characteristic doublet of the Ta 4f_7/2_ and Ta 4f_5/2_ peaks. Curve fitting reveals that the Ta 4f peak can be modeled by three doublets of different tantalum compounds set with a separation between Ta 4f_7/2_ and Ta 4f_5/2_ of 1.91 eV,^46^ an almost equal full-width half-maximum (, , and ), and a peak area ratio of 4:3. The proposed doublets and their respective binding energies can be attributed to TaN_*x*_ [Ta 4f_7/2_ = 24.0 eV (dark blue line) and Ta 4f_5/2_ = 25.9 eV (light blue line)],^47−55^ TaO_*x*_N_*y*_ [Ta 4f_7/2_ = 25.1 eV (dark green line) and Ta 4f_5/2_ = 27.0 eV (light green line)],^27,48,51,56−58^ and TaO_*x*_ [Ta 4f_7/2_ = 26.4 eV (red line) and Ta 4f_5/2_ = 28.3 (orange line)].^29,48,51,55^ The Ta-oxidation state in the TaO_*x*_ component is Ta^5+^, which corresponds to Ta_2_O_5_. However, it was reported that Ta^+^, Ta^2+^, Ta^3+^, and Ta^4+^ oxidation states also appear in TaO_*x*_ films from 22.7 to 25.4 eV.^59^ Therefore, other tantalum oxides, such as TaO_2_ and TaO, may exist at lower binding energies but are indistinguishable from the TaN_*x*_ and TaO_*x*_N_*y*_ components shown in Figure 1c. The experimental data of the Ta 4f peak are represented by the magenta line, and the fitting envelope is represented by the black line. These results suggest that tantalum can be bonded with nitrogen, oxygen, or both simultaneously.

Figure 1d depicts the XPS spectrum from −4 to 3 eV. The detection of electronic states above the Fermi level (*E*_F_) reveals the metallic nature of the compound at room temperature. Additionally, in the electronic properties description (Section 3.6), a detailed discussion of the contributions to the density of states (DOS) is provided.

The quantitative analysis of the Ta 4f, N 1s, and O 1s high-resolution peaks reveals that the elemental composition of the thin film in atomic percentage corresponds to 55% Ta, 25% N, and 20% O, which can be represented approximately by the formula TaN_0.45_O_0.36_, showing that oxygen atoms are replacing the nitrogen atoms. The relative sensitivity factors used to calculate the thin film’s elemental concentration were 4.82, 3.80, 1.80, and 2.93 for Ta 4f_7/2_, Ta 4f_5/2_, N 1s, and O 1s, respectively, as reported by Scofield.^60^ The chemical composition of the material is homogeneous along the 35 nm thickness of the film, since one oxide monolayer at the surface under such UHV conditions can form in a time (τ) of 2000.13 s,^61^ and the *in situ* XPS analysis was performed immediately after deposition.

On the other hand, it is important to remark that adventitious carbon was not found in the samples, which proves that the UHV system was working correctly and that oxygen was incorporated throughout the material during the PLD process, not only at the surface. This fact leads to the conclusion that the source of O contamination is the residual gases inside the growth chamber. If the thin film were air-exposed and analyzed by *ex situ* XPS, a signal of C 1s corresponding to adventitious carbon would be found at the sample surface.

### 3.2 Crystal Structure

**Figure 2:**
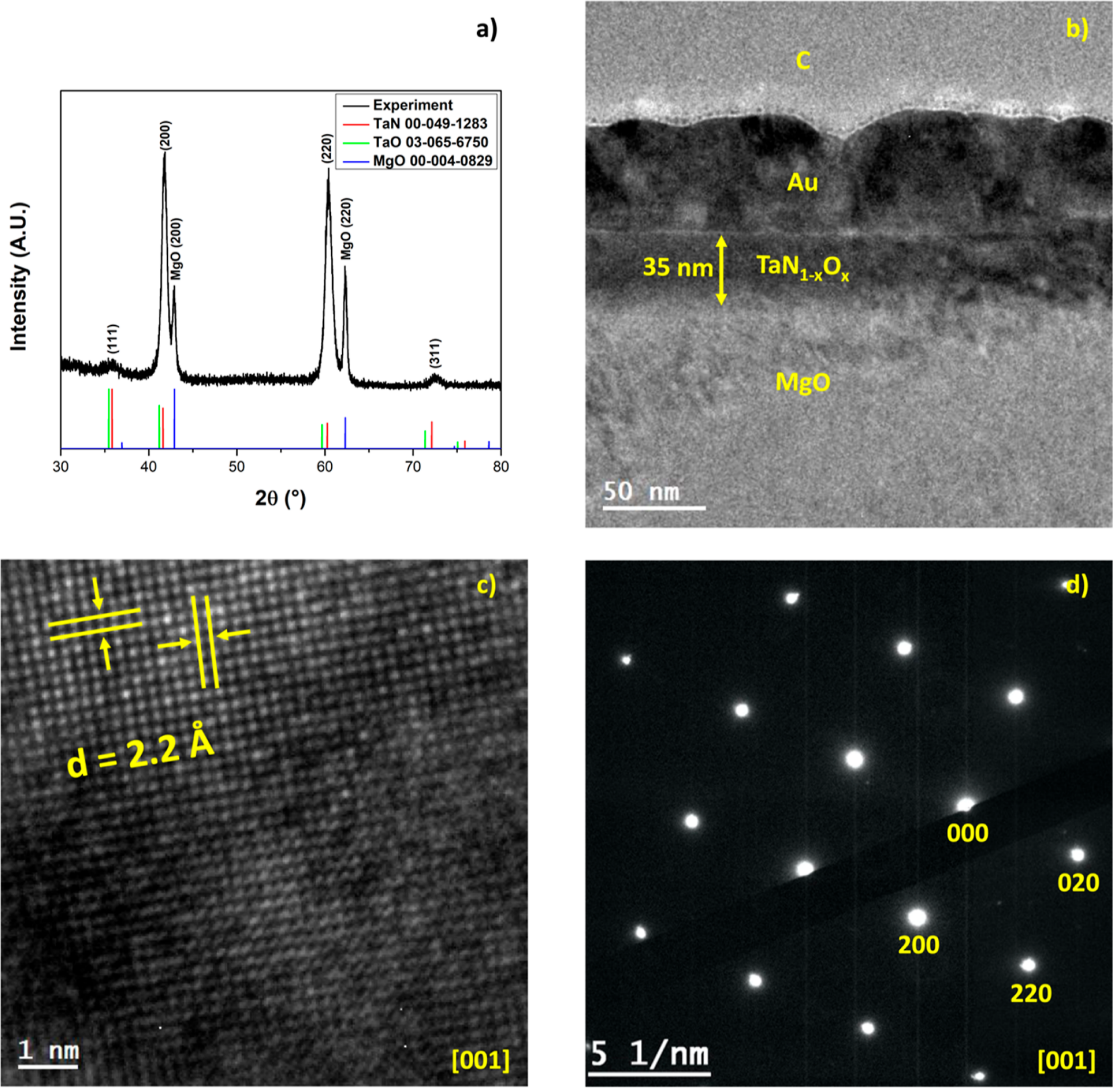
Crystal structure of the thin film by XRD and TEM. (a) XRD pattern, (b) low-magnification micrograph, (c) high-magnification micrograph, and (d) electron diffraction pattern.

Figure 2a depicts the XRD pattern, plotted in the 2θ range from 30 to 80°, for the TaN sample. Several peaks appear in the diffractogram, revealing a polycrystalline film. The peaks at 42.850 and 62.290° correspond to the (200) and (220) planes of the MgO substrate, respectively, as confirmed in the 00-004-0829 crystallographic card. The remaining peaks, related to the thin film, correspond to the (111), (200), (220), and (311) planes, are indexed using the 00-049-1283 (FCC TaN) card and the 03-065-6750 (FCC TaO) card, which present peaks at 35.831, 41.606, 60.258, and 72.125° for TaN and at 35.470, 41.187, 59.660, and 71.364° for TaO, respectively. In the experiment, the (111), (200), (220), and (311) peaks appear at 35.415, 41.845, 60.415, and 72.415°, respectively; those peaks are attributed to both TaN and TaO. The values obtained in the experiment are similar to those reported by the previously mentioned ICDD cards, so the presence of the δ-TaN_1–*x*_ and γ-TaO phases in the thin film is verified. These results agree with Cristea *et al.*, who reported that both FCC phases δ-TaN_1–*x*_ and γ-TaO can coexist in TaO_*x*_N_*y*_ thin films.^20^

Obtaining the δ-TaN_1–*x*_ phase by PLD is possible because when nitride is formed, the Ta–N system is in a state of high excitation produced by the laser energy. The relaxation of the Ta–N system occurs in an extremely short time that does not allow the atoms to rearrange themselves into another structure. Therefore, the desired crystalline phase is obtained even though it is metastable. The γ-TaO phase appears due to the residual gases contained inside the growth chamber. It is worth mentioning that although the Ta_2_O_5_ phase is the most stable and known for tantalum oxide, γ-TaO shares the same space group *Fm*3̅*m* with δ-TaN_1–*x*_ and has a reported lattice parameter of 4.44 Å.^17,62^

Transmission electron microscopy is used to characterize the film’s local crystalline growth, as shown in Figure 2b–d. The micrographs and diffraction patterns of the thin film cross-section contain information about the local crystal structure of the material and its interplanar spacing. Figure 2b displays a micrograph recorded at low magnification of the cross-section of the sample prepared by FIB for TEM, showing the MgO substrate, the 35 nm TaN_1–*x*_O_*x*_ thin film, and the Au and C layers used to protect the sample from FIB erosion.

A high-resolution micrograph of the TaN_1–*x*_O_*x*_ thin film cross-section is shown in Figure 2c; the micrograph corresponds to a region near the edge of the film. The local growth follows the substrate direction and presents an FCC structure with an interplanar spacing of 2.2 Å, associated with the (200) and (020) planes. Figure 2d shows the thin film’s indexed selected area electron diffraction (SAED) pattern. An FCC structure is confirmed, and the (200), (020), and (220) planes are associated with it. The [001] beam direction is labeled in both Figure 2c,d.

The FCC crystalline phases of TaN and TaO are evident in the X-ray diffractogram, see Figure 2a. No diffraction of more stable phases, such as monoclinic TaON or orthorhombic Ta_2_O_5_, appears; these phases are absent in the TEM micrographs and SAED patterns as well. In the low-magnification micrographs, it is observed that the film thickness is ∼35 nm. Since our sample was grown at 850 °C, the obtained preferential orientation is the (200) reflection (see Figure 2a), in agreement with the behavior reported by Elangovan *et al.*, who showed that above 600 °C, the preferential orientation changes from (111) to (200).^63^

### 3.3 Superconductivity Measurements

**Figure 3:**
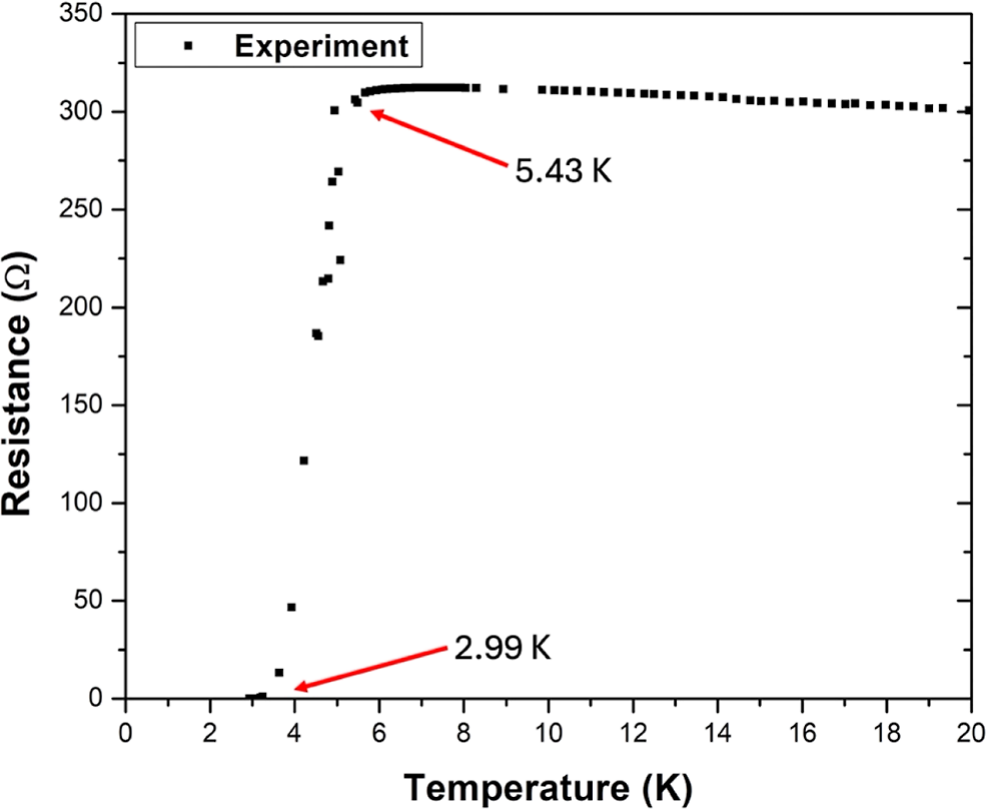
Resistance *vs* temperature curve of the thin film measured by the four-point probe method. The onset is at 5.43 K, and the zero-resistance point is at 2.99 K.

Figure 3 shows the resistance *vs* temperature curve of the tantalum oxynitride thin film; the area of interest is plotted from 0 to 20 K. The film presents superconductivity at a *T*_c_ of 2.99 K; its resistance decreased abruptly from 306.11 Ω (5.43 K) to 0 Ω (at 2.99 K). Although magnetic susceptibility was not measured, the abrupt drop in the curve allows us to infer that the sample is superconducting with a measured zero resistance at 2.99 K; to confirm that value, the instrument measured 1413 points in that zone. Some authors report the *T*_c_ at the onset of the transition.^9^ In our case, the onset is located at 5.43 K, reaching zero resistance at 2.99 K, so the transition has a width of 2.44 K. The superconductivity of the tantalum oxynitride film is a consequence of the presence of the FCC structure, as previously discussed in the literature for tantalum nitride.^2^ The stoichiometry has less weight than the crystalline structure in the superconductivity since the film does not lose its superconducting character, even when a considerable amount of oxygen impurities (20 at. %) appear. Also, superconductivity was recently found in bulk FCC TaO with *T*_c_ = 6.20 K,^17^ so it is expected that the possible TaO regions formed in the thin film are superconducting as well. The consequence of having random oxygen impurities in the sample is that the film *T*_c_ is lower than that reported by Reichelt *et al.*, where they found a *T*_c_ = 10.8 K.^5^ However, our measured *T*_c_ is within the range of reported values for TaN thin films by other authors as ∼4.31–8.15,^8^ 5.1–10.8,^5^ 5.9–7,^9^ 6–8,^11^ and 0.5–6.4 K;^10^ or for the bulk TaN *T*_c_ = 6.5 K.^16^

In other words, the oxygen contained in the sample prevents the tantalum nitride films from reaching their maximum possible *T*_c_. Notice that even at highly controlled growth conditions, oxygen impurities appear. The differences in the reported *T*_c_ indicate that the role of oxygen impurities in superconductivity performance still needs to be discussed appropriately in the literature. Also, it is evident that TaN is highly reactive to oxygen, and even under UHV, the residual oxygen would be present in the thin films. To corroborate our assumptions, we simulate—at the atomic scale—the oxygen capacity to incorporate into the FCC TaN lattice.

### 3.4 Atomic-Scale Analysis of Oxygen Incorporation into TaN

In this section, using DFT calculations, we investigate the presence of oxygen in the TaN structure. The calculations use TaN as a reference. The highest O content is modeled as the TaO structure. TaN and TaO are isostructural. The calculated lattice parameter for TaN is 4.42 Å with a Ta–N bond length of 2.21 Å, and the cell parameter for TaO is 4.51 Å with a Ta–O bond length of 2.25 Å, in agreement with previous experimental reports.^6,17^ A 2 × 2 × 2 periodicity is employed to study different oxide contents. The models contain 64 atoms distributed in 32 Ta atoms and 32 atoms that can be N, O, or a combination of both.

Table 1 summarizes the cell parameters for each considered stoichiometry. As the oxygen amount increases, the TaN_1–*x*_O_*x*_ cell parameter expands until it reaches an oxygen content of *x* = 0.50. After that, the cell parameter contracts to the TaO lattice parameter. The structural models of TaN, TaN_0.5_O_0.5_, and TaO are depicted in Figure 5; the remaining models are shown in the Supporting Information section. Oxygen is randomly distributed to avoid initial clustering. In the experiment, few oxygen atoms were available in the UHV chamber. As discussed in the Methods section, insufficient oxygen is available to get clustering. Also, experimental evidence suggests that oxygen enters at the N sites in the TaN lattice.

**Table 1:** Oxygen Content in the TaN_1–*x*_O_*x*_ Supercells

model	number of O atoms	% O	lattice parameter (Å)
TaN	0	0.00	8.84
TaN_0.97_O_0.03_	1	1.56	8.85
TaN_0.94_O_0.06_	2	3.13	8.85
TaN_0.87_O_0.13_	4	6.25	8.86
TaN_0.81_O_0.19_	6	9.38	8.90
TaN_0.75_O_0.25_	8	12.50	8.93
TaN_0.50_O_0.50_	16	25.00	9.03
TaO	32	50.00	9.01

**Figure 4:**
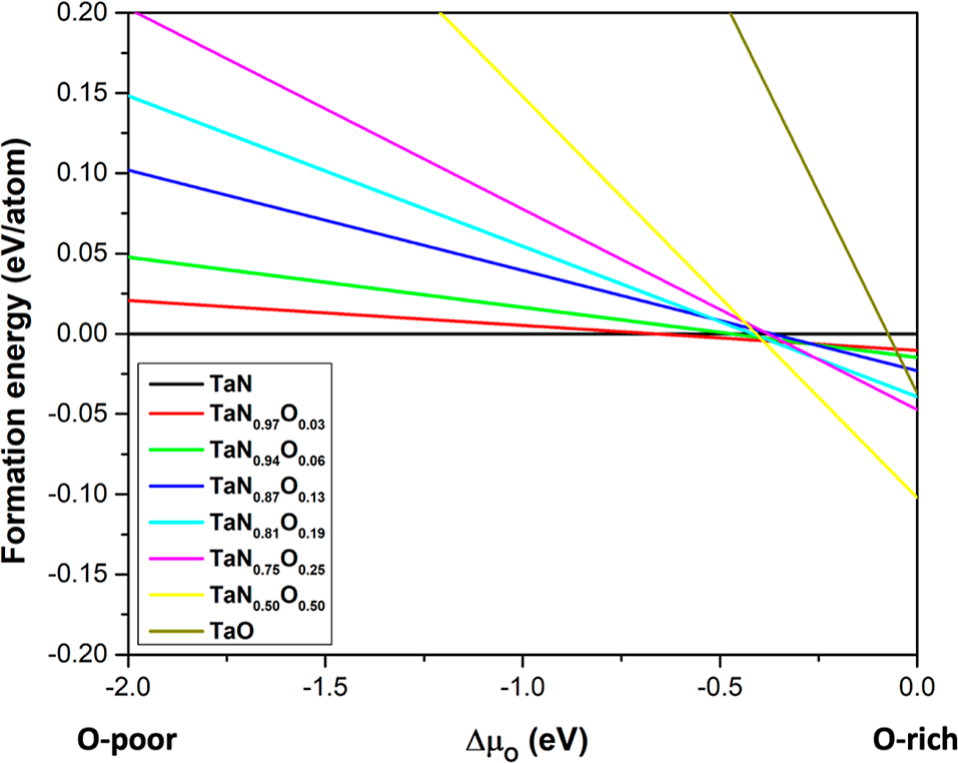
Formation energy *vs* oxygen chemical potential from O-poor to O-rich conditions.

**Figure 5:**
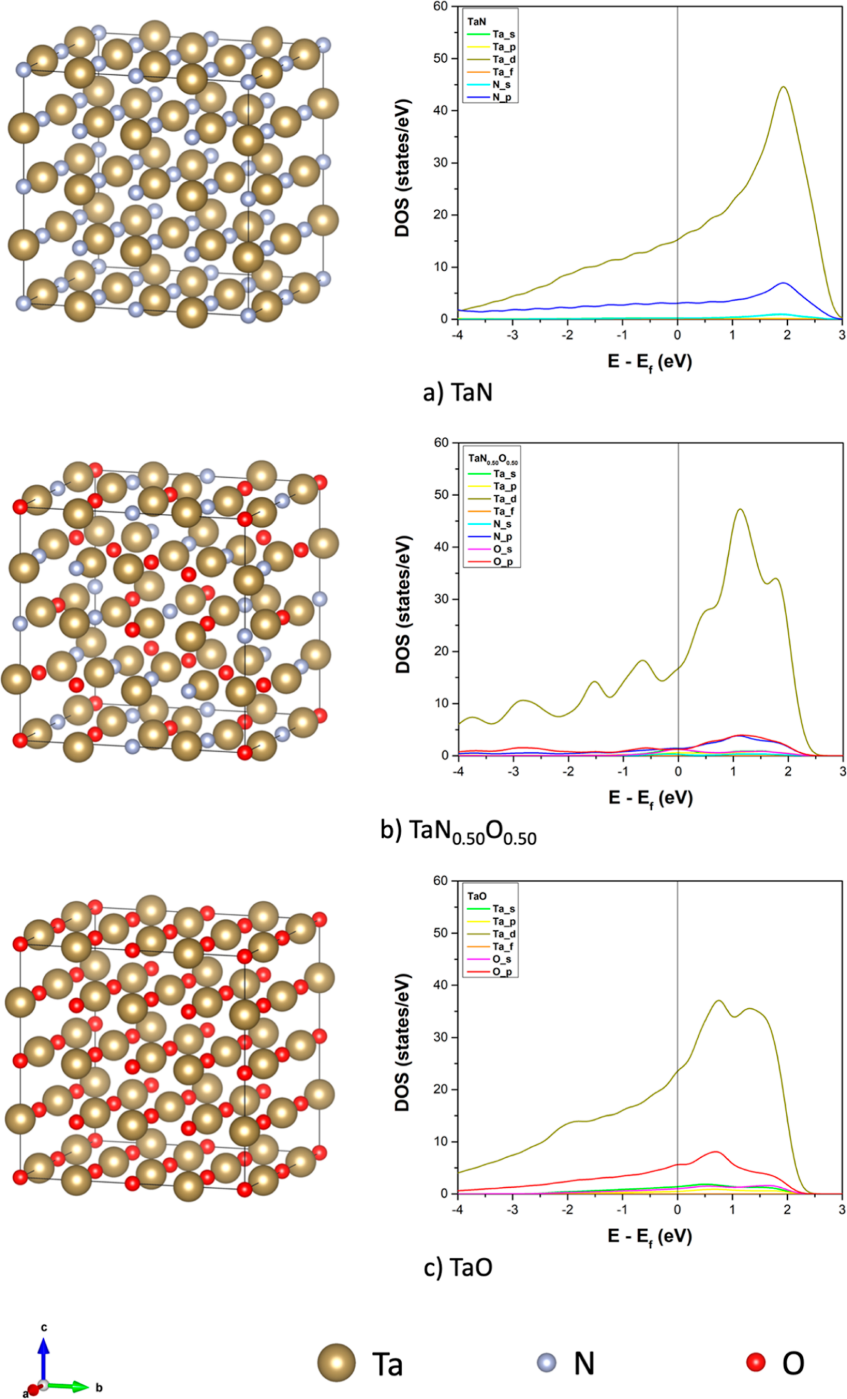
Structural models and projected DOS from −4 to 3 eV of (a) TaN, (b) TaN_0.50_O_0.50_, and (c) TaO. The Fermi level is set to 0 eV. The graphs show the contribution of the s and p orbitals of N and O and the contribution of s, p, d, and f orbitals of Ta.

### 3.5 Thermodynamic Stability of the Tantalum Oxynitrides

To analyze the viability of oxygen replacing nitrogen atoms in the TaN lattice, we evaluated the DFE. Figure 4 depicts the formation energy *vs* oxygen chemical potential from O-poor to O-rich conditions. Stable models must have lower energy than the reference (negative DFE); otherwise, they are unstable. In Figure 4, the left part of the graph corresponds to O-poor conditions, while the right part corresponds to O-rich conditions. Note that the bare TaN is the most favorable model under O-poor and intermediate conditions, which is expected since there is a lack of oxygen under O-poor conditions.

On the other hand, all the substitution models are viable under O-rich conditions—such energies demonstrate the spontaneous oxygen incorporation—, even the TaN_0.97_O_0.03_ composition, which has the lowest oxygen content. The stability of each model increases as oxygen atoms replace nitrogen from the TaN lattice until they reach the TaN_0.50_O_0.50_ composition. After that, it is expected to decrease in stability until reaching TaO. Note that TaO is included as a limit case. However, in the experiment, such oxygen quantities are not expected to be observed since we work with an UHV system. As mentioned in the Methods section, the oxygen in TaN may be due to the residual gases inside the growth chamber. It should be noted that the compositions studied in this work are less stable than hexagonal TaN, monoclinic TaON, and orthorhombic Ta_2_O_5_ (the most stable phases of the three compounds). Nevertheless, FCC TaN can be induced using a physical vapor deposition technique on MgO, Si, or sapphire substrates.^5,9,11,12^

Our DFE analysis evinces that even at low oxygen concentrations, the TaN tends to oxidize, so tantalum oxynitride must always be considered when discussing the superconductivity measurements, which has been overlooked in the existing literature.

As another piece of evidence about the spontaneous formation of tantalum oxynitride, some authors have reported that ε-TaN may not exist as a binary nitride.^64^ Still, it stabilizes with oxygen atoms, a hypothesis that matches our results for δ-TaN_1–*x*_.

### 3.6 Electronic Properties

To analyze the behavior of the most stable tantalum oxynitride structure, we investigated the electronic properties of the TaN_0.5_O_0.5_ model and compared it with the TaN and TaO models by calculating its DOS. The Fermi level is the energy reference. A metallic behavior emerges and agrees with the electronic states obtained by XPS measurements, see Figures 1d and 5b. The results for the three systems are depicted in Figure 5.

Regarding the bare TaN, our DOS agrees with the report by Stampfl and Freeman,^65^ where the main contribution to the Fermi level comes from the Ta d orbitals. Similar behavior is observed for TaN_0.50_O_0.50_, where the Ta d orbitals mainly contribute to the DOS around the Fermi level. In contrast, the N p and O p orbitals contribute almost equally. In the case of TaO, the main contribution at the Fermi level comes from the Ta d orbitals, followed by the O p contributions.

**Figure 6:**
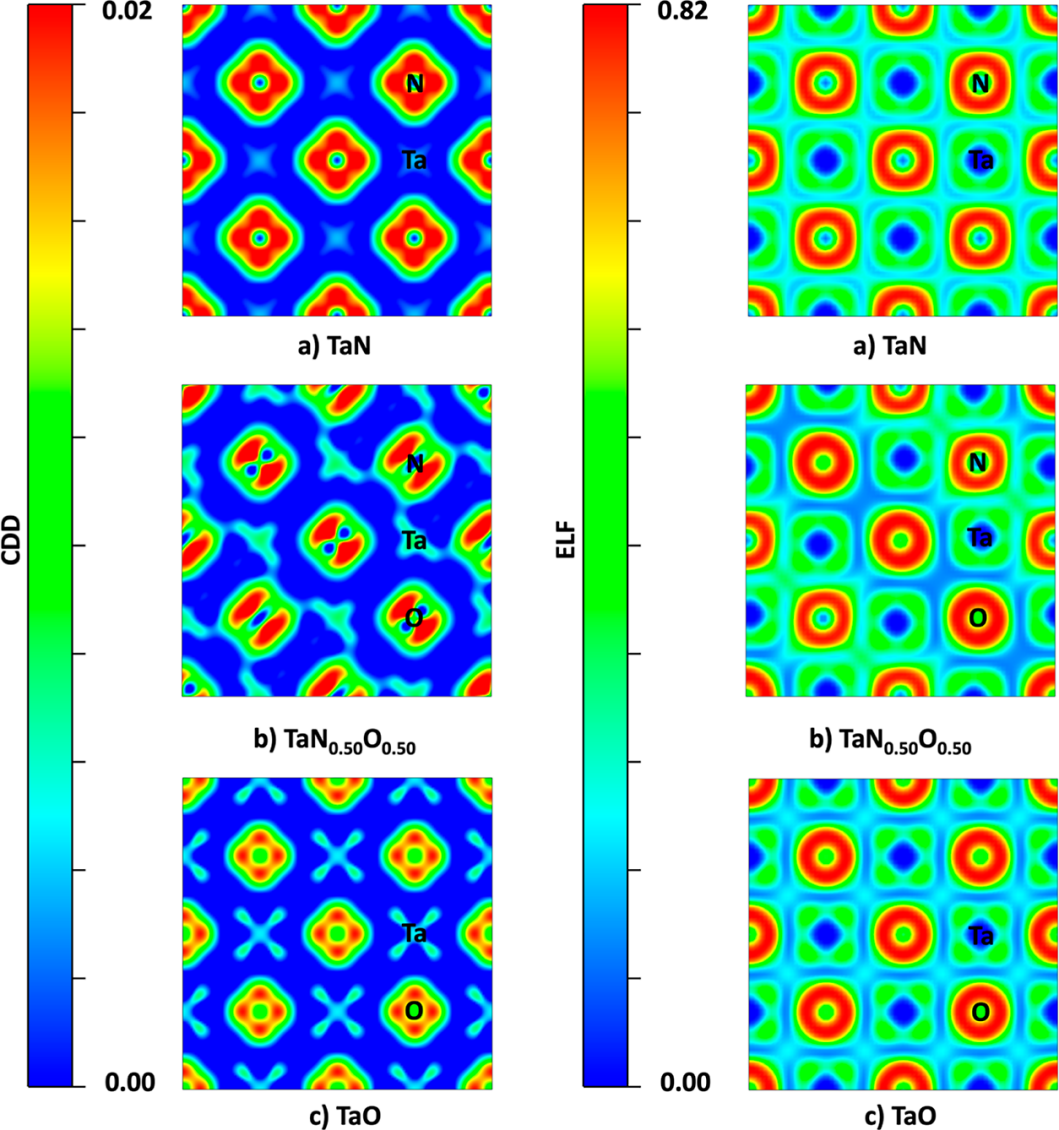
A 2D slice view of the CDD from 0 to 0.02 and the ELF from 0 to 0.82 of (a) TaN, (b) TaN_0.50_O_0.50_, and (c) TaO at the (010) plane.

To elucidate the bonding type in the pristine and oxidized systems, we plot the charge density difference (CDD) and the electron localization function (ELF) at the (010) plane. An RGB scheme was used in both cases. The CDD of (a) TaN, (b) TaN_0.5_O_0.5_, and (c) TaO are shown in the left panel of Figure 6. Blue indicates charge depletion, while red indicates charge accumulation. In all cases, the Ta atoms show a depletion of the electronic density. In contrast, N and O atoms exhibit charge accumulation, suggesting charge transfer between neighboring atoms and an ionic nature.

**Figure 7:**
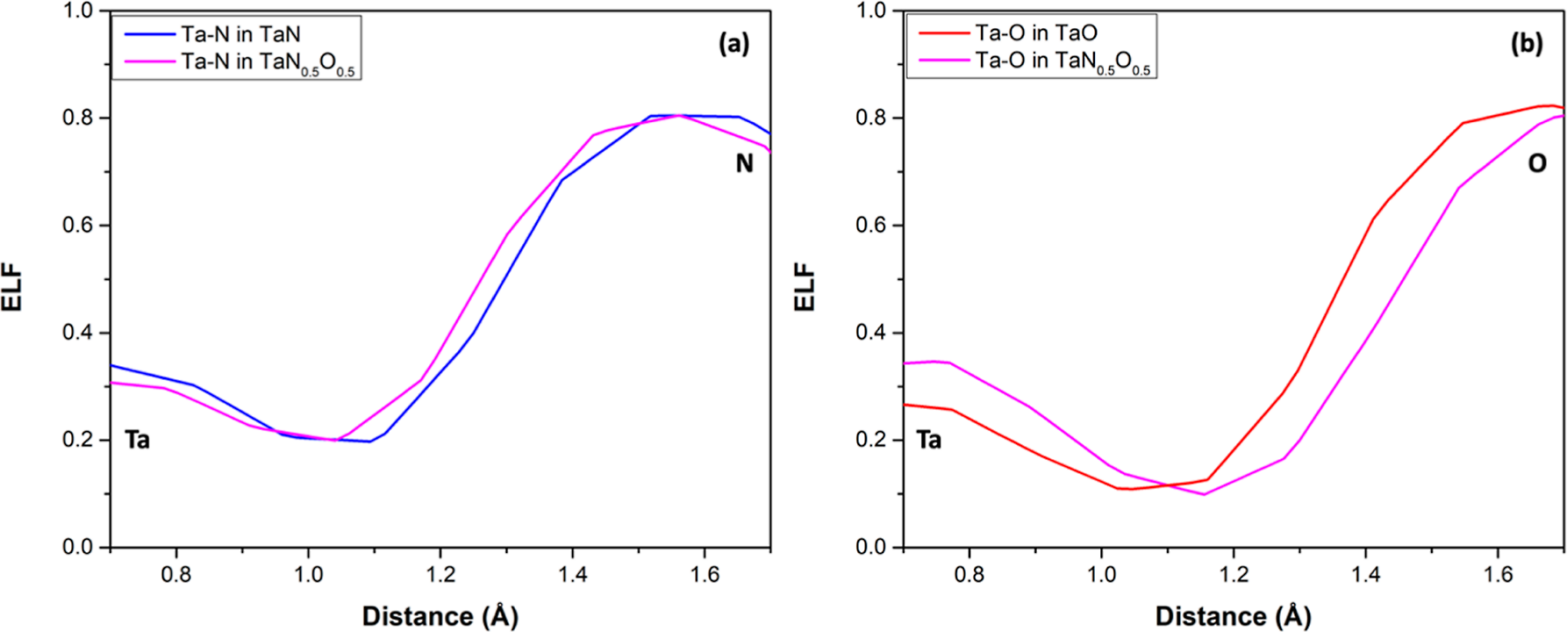
Line profiles of the ELF of the (a) Ta–N and (b) Ta–O bonds of the TaN, TaN_0.5_O_0.5_, and TaO supercells.

To further confirm the ionic character of the bonds in the structures, ELF density maps for the same systems are plotted in the right panel of Figure 6. The blue color suggests a low probability of finding electron accumulation; otherwise, it is characterized by red. Tantalum nitride, oxynitride, and oxide show similar behavior since the main probability of finding electrons is around the electronegative atoms. Also, electron depletion is observed in the middle point between Ta–O and Ta–N, which suggests an ionic interaction. This fact is corroborated by the ELF line profiles shown in Figure 7, where the ionic nature of both interactions is easy to observe; however, the Ta–O provides the most ionic-type interaction since, at the middle point of the bond, less electron population is observed.

## 4 Conclusions

Superconducting tantalum nitride was synthesized by reactive PLD on an FCC MgO substrate. It showed zero electrical resistance at *T*_c_ = 2.99 K. The film, with a thickness of 35 nm, exhibited an FCC structure, as confirmed by XRD and TEM. No secondary phases were observed; however, elemental analysis by *in situ* XPS evinces the presence of stable oxygen impurities amounting up to 20 at. %. The oxygen source is the residual gases inside the growth chamber. Using DFT calculations and thermodynamic criteria, we obtained evidence that oxygen can occupy the N sites of the crystal without structural modifications. Our experimental and theoretical findings support the idea that TaN does not exist in pure form but as a tantalum oxynitride. Therefore, it is important to consider the presence of oxygen in the superconductor and how it modifies its properties, a fact not considered previously in the literature and possibly the main reason for the different critical temperatures measured in tantalum nitride materials.

## Supporting Information Available

Structural model and projected DOS from −4 to 3 eV of TaN_0.97_O_0.03_ (Figure S1), TaN_0.94_O_0.06_ (Figure S2), TaN_0.87_O_0.13_ (Figure S3), TaN_0.81_O_0.19_ (Figure S4) and TaN_0.75_O_0.25_ (Figure S5) (PDF)
