# Supplementary material for: Stable Oxygen Incorporation in Superconducting TaN: An Experimental and Theoretical Assessment
Source: ACS Omega. 2024 Aug 1;9(32):35069–79. doi: 10.1021/acsomega.4c05310 (PMC11325398; doi:10.1021/acsomega.4c05310)
Supplement: Supplementary file 1 — ao4c05310_si_001.pdf [file ao4c05310_si_001.pdf]

Supporting information

Stable oxygen incorporation in superconducting TaN: An experimental and theoretical assessment

Victor Quintanar-Zamora [a,b], Michelle Cedillo-Rosillo [a,b], Oscar Contreras-López [b], Carlos Antonio Corona-Garcia [b], Armando Reyes-Serrato [b], Rodrigo Ponce-Pérez [b], Jonathan Guerrero-Sanchez [b], and Jesús Antonio Díaz [b*]

[a] Centro de Investigación Científica y de Educación Superior de Ensenada, Posgrado en Nanociencias, Ensenada, Baja California, 22860, México
[b] Universidad Nacional Autónoma de México, Centro de Nanociencias y Nanotecnología, Ensenada, Baja California, 22860, México

[*] Corresponding author: olaf@ens.cnyn.unam.mx

The optimized supercells and projected density of states calculations of the $TaN_{1-x}O_x$ models containing different amounts of oxygen atoms are shown below. Dark yellow atoms represent Ta, blue atoms represent N, and red atoms represent O.

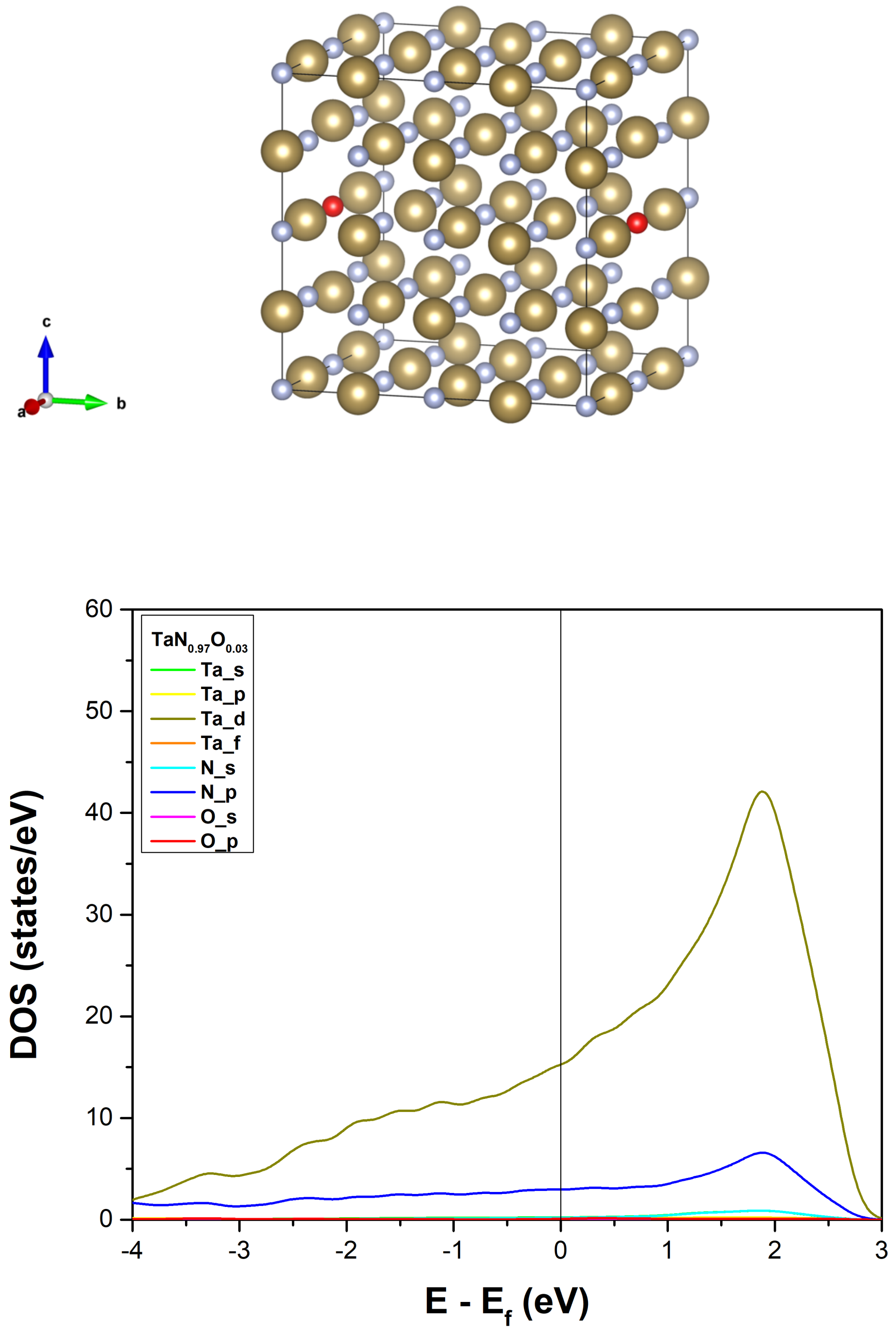

Figure S1. Structural model and projected density of states from -4 to 3 eV of $TaN_{0.97}O_{0.03}$.

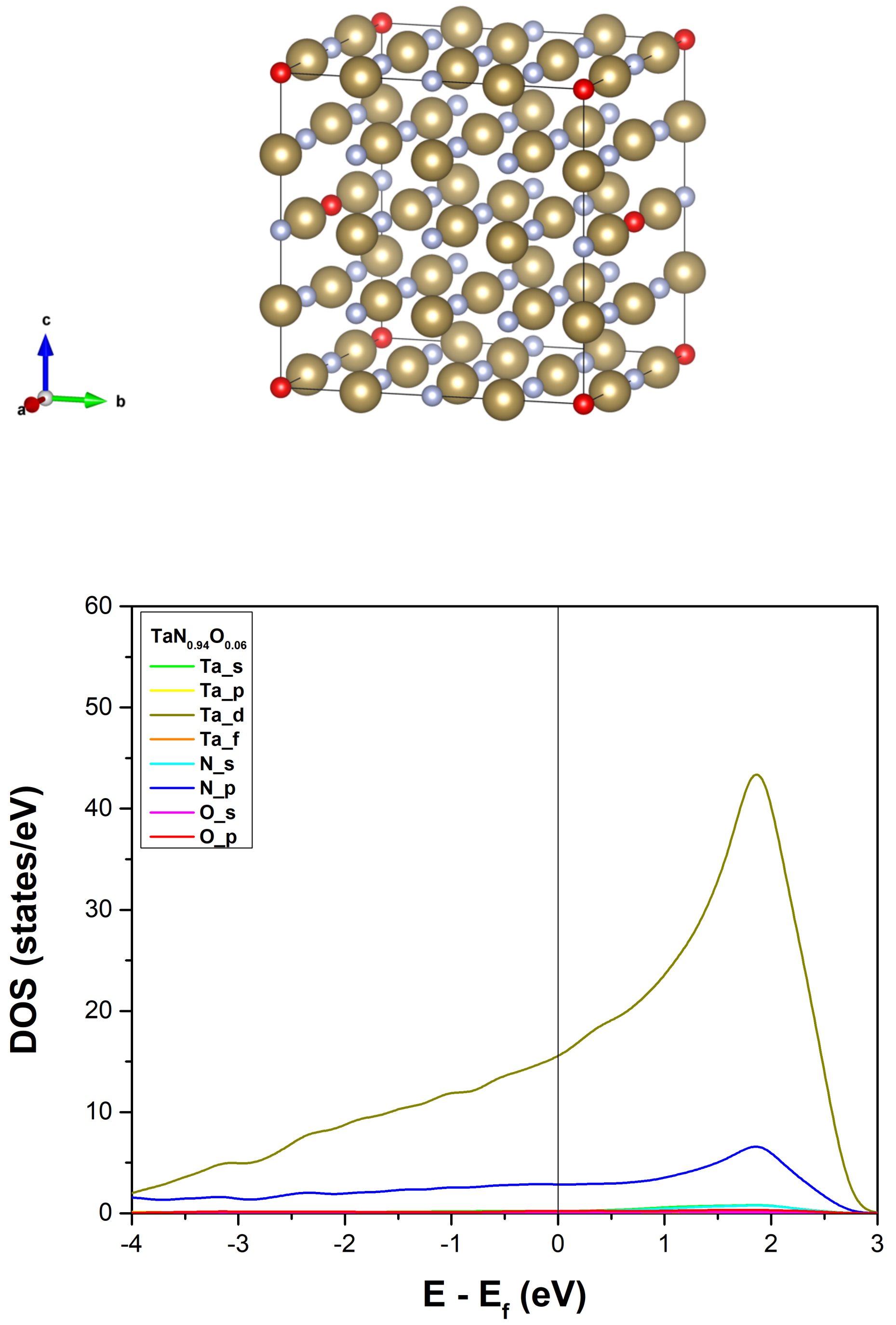

Figure S2. Structural model and projected density of states from -4 to 3 eV of $TaN_{0.94}O_{0.06}$.

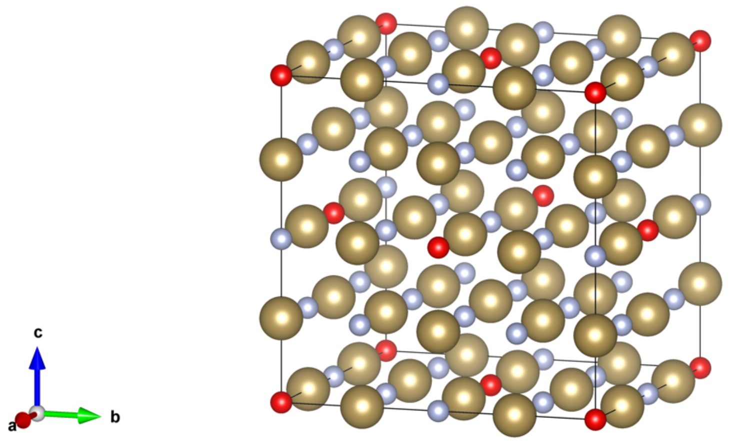

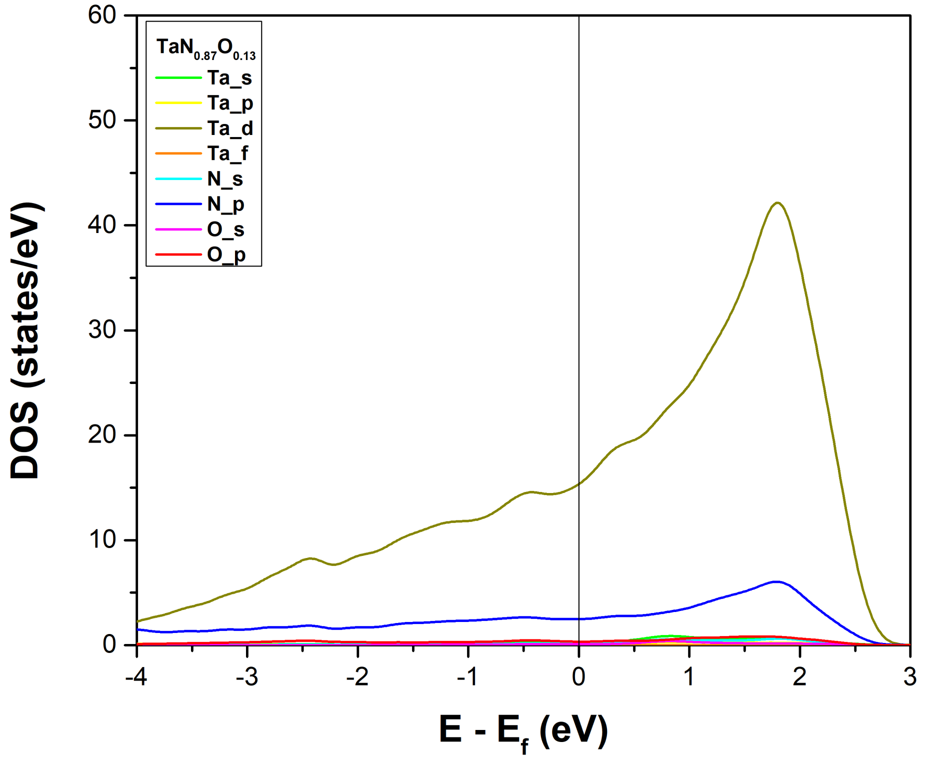

Figure S3. Structural model and projected density of states from -4 to 3 eV of $TaN_{0.87}O_{0.13}$.

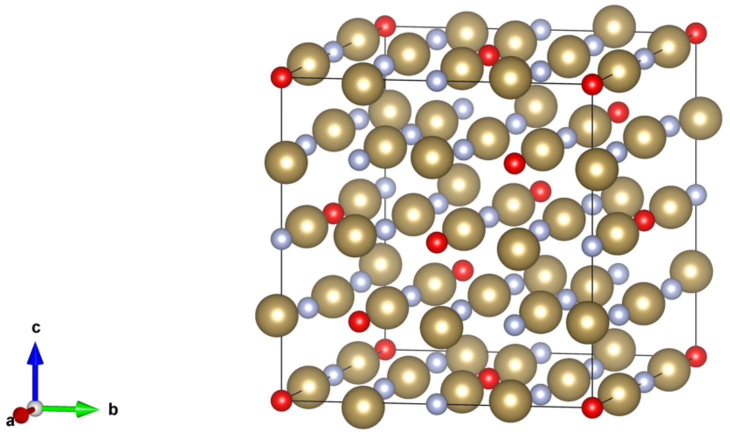

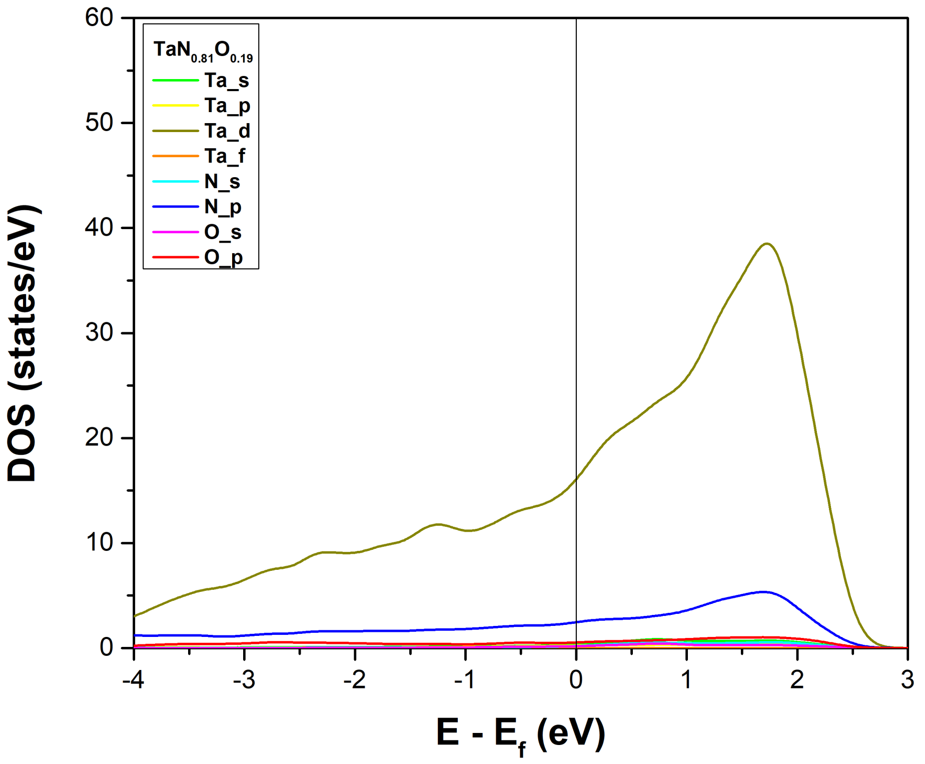

Figure S4. Structural model and projected density of states from -4 to 3 eV of $TaN_{0.81}O_{0.19}$.

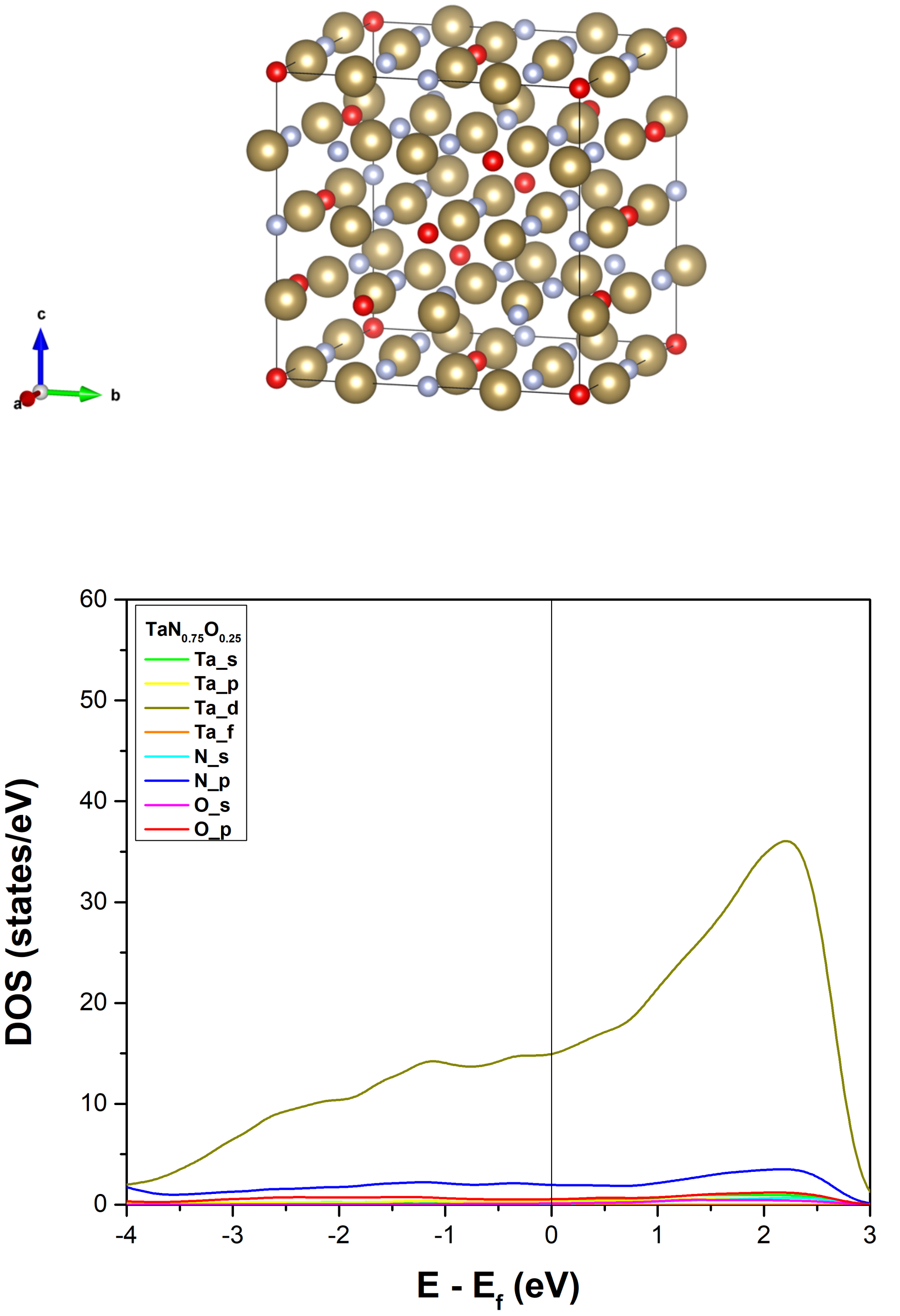

Figure S5. Structural model and projected density of states from -4 to 3 eV of $TaN_{0.75}O_{0.25}$.
